# Signal Novelty Detection as an Intrinsic Reward for Robotics

**DOI:** 10.3390/s23083985

**Published:** 2023-04-14

**Authors:** Martin Kubovčík, Iveta Dirgová Luptáková, Jiří Pospíchal

**Affiliations:** Department of Applied Informatics, Faculty of Natural Sciences, University of Ss. Cyril and Methodius, J. Herdu 2, 917 01 Trnava, Slovakia; iveta.dirgova.luptakova@ucm.sk

**Keywords:** anomaly detection, autoencoder, signal processing, intrinsic reward, robotics, exploration

## Abstract

In advanced robot control, reinforcement learning is a common technique used to transform sensor data into signals for actuators, based on feedback from the robot’s environment. However, the feedback or reward is typically sparse, as it is provided mainly after the task’s completion or failure, leading to slow convergence. Additional intrinsic rewards based on the state visitation frequency can provide more feedback. In this study, an Autoencoder deep learning neural network was utilized as novelty detection for intrinsic rewards to guide the search process through a state space. The neural network processed signals from various types of sensors simultaneously. It was tested on simulated robotic agents in a benchmark set of classic control OpenAI Gym test environments (including Mountain Car, Acrobot, CartPole, and LunarLander), achieving more efficient and accurate robot control in three of the four tasks (with only slight degradation in the Lunar Lander task) when purely intrinsic rewards were used compared to standard extrinsic rewards. By incorporating autoencoder-based intrinsic rewards, robots could potentially become more dependable in autonomous operations like space or underwater exploration or during natural disaster response. This is because the system could better adapt to changing environments or unexpected situations.

## 1. Introduction

Reinforcement learning is a well-known approach for teaching robots in simulated environments. In this method, the robot is considered as an agent that receives information in the form of observations about itself and its environment. The agent—simulated robot performs actions in the environment and receives a reward signal as feedback. The learning algorithm then tries to predict the ideal actions that will lead the agent to success, based on the received reward signal. However, there are simulations where the reward signal is insufficient for the agent to complete the overall task at hand. This happens when the rewards are sparse, meaning that the agent only receives a high reward after successfully completing the task, or a negative reward after a significant failure. In such cases, an intrinsic reward needs to complement the reward signal from the environment.

Intrinsic reward-based control refers to a method of controlling robots that involves giving them internal rewards for performing certain actions or achieving certain goals. Ideally, these rewards are not explicitly defined by the programmer but instead are learned by the robot through trial and error. The idea is that the robot will develop its own goals and motivations based on its experiences, which will allow it to perform tasks efficiently and adapt to new situations easily.

Existing control techniques, on the other hand, are based on well-established principles of control theory, which have been developed over many years. These techniques typically involve setting up a feedback loop between the robot and its environment, in which the robot measures its performance and adjusts its actions accordingly.

Intrinsic reward-based control is a relatively new approach that has gained popularity in recent years, while existing control techniques have been developed over many decades and are widely used in robotics.

The intrinsic reward, as it is understood in this paper, is a supplementary form of feedback that conveys additional information about the agent’s state in the environment, such as the frequency of visited states in the robot’s state space. The agent is compelled to seek out and respond positively to newly discovered states that represent anomalies in its observation. As the states are visited more frequently, they become less anomalous, and therefore, the reward for visiting them decreases over time. A similar approach is used in [1], where the reward increases if the predicted next state is different from the actual next state. However, in cases where the environment is sufficiently stochastic, the agent cannot predict the next state accurately, resulting in a problem known as Noisy TV [2]. To overcome this, the deep neural network can memorize the states from the environment instead of learning the environment’s dynamics.

Many methods have been proposed in the past to supplement the rewards generated by the environment. One of the earliest approaches, the Counting by Density Model [3], used a probabilistic model to express a pseudo-count metric based on state visitation counts in the agent’s state space. Another method, Counting after Hashing, transformed high-dimensional states into hash codes and stored their visitation frequency [4]. Curiosity-based approaches, such as Intelligent Adaptive Curiosity [5], Intrinsic Curiosity Module [1], and Variational information maximizing exploration [6] aimed to learn the dynamics of the environment by predicting subsequent states. However, these methods struggled to handle stochastic environments. Directed Outreach Reinforcement Action-Selection [7] and Random Network Distillation [2] used E-values (similar to a Q-value) and memorization, respectively, to address this issue. Random Network Distillation uses a pair of models where one is just randomly initialized, and the other model learns to predict the same features as generated by the random model. Generative Adversarial Network-based Intrinsic Reward Module learned the distribution of observed states and rewarded the agent for exploring unexplored states [8]. The GAN model is trained to produce samples that closely resemble real-world states, while simultaneously training the encoder to map observed states onto a latent noise space. Subsequently, the generator utilizes the regenerated latent noise space to generate new observed states. The intrinsic reward is determined by calculating the mean squared error between the original states and the regenerated states. If the states are unfamiliar, the Generator block is unable to accurately re-generate them, leading to an increase in deviation. An advantage of this approach is that the discriminator’s evaluation of previously unseen states becomes irrelevant. Hyperparameter tuning was critical to the success of this method [8]. A different approach, called Occupancy-Reward-Driven Exploration [9], has been applied in robotics to explore uncharted territories within the state space. In this technique, an occupancy map is utilized to acquire information about the environment through sensors such as a laser sensor. The occupancy map comprises occupancy probability values, where the likelihood of an obstacle is represented by a probability value. A higher value is assigned to regions with a greater level of confidence in obstacle detection, while an unknown region has a probability of 0.5, and a probability of 0 signifies the absence of obstacles. The robot’s reward is then determined by the number of new segments discovered within the occupancy map at each time step. This approach can also improve the robot’s power efficiency [9].

Intrinsic rewards have already been employed in a wide range of applications of deep neural network robot control. One instance is the use of intrinsic rewards to maintain a safe distance between a robot arm and a human operator [10]. In another application, intrinsic rewards were utilized to facilitate collaboration between multiple autonomous robots. This was achieved through a combination of curriculum-based learning, PPO algorithm, and deep learning of convolutional neural network to process multi-channel visual inputs [11]. In [12], an image was used as a state space for curiosity-driven navigation strategy of mobile robots. Moreover, curiosity contrastive forward dynamics model using efficient sampling for visual input was implemented in [13]. Furthermore, intrinsic rewards were employed alongside extrinsic rewards to simulate robotic hand manipulation in [14]. Next, intrinsic rewards were used in [15] to assist robots in docking charging stations by providing visual identification. In [16], the goal of the penalty-based surprise intrinsic reward was gentle object manipulation. Deep CNN-based method found their application even in wire making [17].

The main inspiration for the present work was the approach which solved Atari games through neural networks [18]. While both the objective of this work to address the sparse reward problem [2] and the approach in [18] share similarities, it’s worth noting that in Atari games, the only signal available was an image of the game screen, and the player didn’t directly control a robot equipped with various sensors.

Unlike the methods mentioned earlier, this paper focuses on utilizing the AutoEncoder architecture as an anomaly detector in the signal. This approach can compute intrinsic rewards from anomalies, providing information about a new, previously unseen state in the environment where the agent is moving. The principles employed in this study are directly linked to earlier research on anomaly detection in data mining. Previous uses of intrinsic reward for anomaly detection only involved labeling datasets or simpler tasks that were unrelated to robot control from the signal [19,20,21,22]. However, in one case, anomaly detection was used to identify subgoals when solving a complex problem [23]. In this study, novelty detection is used for simulated robot movement. In a deep learning neural network, the AutoEncoder approach is appropriate for memorizing already explored states. For new, previously unseen states, the Decoder block will inaccurately reconstruct the reduced signal, which can be measured as novelty detection for the intrinsic reward. Therefore, high intrinsic reward originates from an anomaly that the Decoder block cannot transform correctly into the original state. As a result, the agent is compelled to explore the environment to locate unexplored regions and states.

The agent’s objective was not to complete the assigned task (even though the test ended when the task was completed), but rather to increase its score by exploring new states in the environment that it had not yet encountered. This approach ensured that the agent did not revisit already seen states and prioritized maximizing the score by visiting unexplored states.

Current state-of-the-art techniques for optimizing agent performance use a combination of non-episodic and episodic [24] approaches, where a neural network training parameterizes a spectrum of policies ranging from highly exploratory to entirely exploitative. Another approach involves suppressing forgetting in a neural network to enhance performance [25].

Our tests on a set of benchmark environments established that the anomalies themselves can guide the agent to successfully complete the task and that the information extracted from them is sufficient for the agent to converge correctly. To the best of our knowledge, there has been no instance of accomplishing successful robotic guidance solely through novelty seeking using an intrinsic function based on an autoencoder.

## 2. Materials and Methods

The following section is divided into three main parts. Firstly, a brief description of the used benchmark set of test environment for simulated robots is provided. Subsequently, the article describes the control architecture of robots, which utilizes deep neural networks incorporating AutoEncoder, and provides an account of how intrinsic rewards are computed and applied.

### 2.1. Benchmark Set of Test Environments for Simulated Robots

The selected test environments belong to the standard tasks solved using reinforcement learning methods. These environments have a continuous state space and a discrete action space. The LunarLander-v2 [26] problem presents a classic rocket trajectory optimization challenge where the objective is to land a lunar lander on a designated landing pad. CartPole-v1 [27] is another problem that involves balancing an inverted pendulum on a motor-driven cart, where the aim is to maintain the pole’s vertical position by moving the cart to the right or left. The Acrobot-v1 problem [28,29] requires the agent to reach a specific height target with a simple 1-joint arm. The system consists of two links, one of which is fixed, and the other is connected to it. The starting position has both links hanging down, and the objective is to apply force to change the angle of the joint so that the free end of the links swings above a target height. In the MountainCar-v0 environment [30], the goal is to drive a car up a hill to reach the target state. Initially, the car is randomly placed at the bottom of a sinusoidal valley, and the car can be accelerated in either direction, but not strongly. The solution requires the utilization of potential energy by driving up the opposite hill.

### 2.2. AutoEncoder Architecture

AutoEncoder is one of the techniques utilized in deep neural networks for identifying anomalies in robotic sensor signals [31]. This approach involves training on previously observed states from the experience replay buffer (RB) [32], and then predicting future states based on those observations. As a result, the AutoEncoder can quickly identify any new, previously unobserved states, allowing the agent to explore its environment and access unvisited states within its state space.

Anomaly detection relies on a combination of exploration and exploitation, utilizing AutoEncoder-based metric learning to measure reconstruction error in the agent’s prediction. The AutoEncoder loss function is calculated as the commonly employed mean squared error:

(1) $$Loss=\frac{1}{n}\sum_{i=1}^{n}{\left[{state}_{i}-{AE\left(state\right)}_{i}\right]}^{2},$$

where *n* represents the number of features in the agent’s state space, the *state_i_* represents the feature of the state that the agent obtains from the environment, and *AE_i_* represents the output of the AutoEncoder model.

Intrinsic reward is calculated as the reconstruction error:

(2) $$err(state)=\sum_{i=1}^{n}{\left[{state}_{i}-{AE\left(state\right)}_{i}\right]}^{2},$$

(3) $$Intrinsic\;reward=ReLU6(\frac{err\left(state\right)-\mu_{e}}{\sigma_{e}}),$$

where *μ_e_* and *σ_e_* are running mean and standard deviation for err(*state*). When computing the usual reconstruction error [33], feature averaging is not taken into account. This is because if there are many sensors and most of them measure typical values while only one sensor exhibits a significant difference, applying averaging could potentially mask this signal, resulting in the loss of the intrinsic reward.

**Figure 1:**
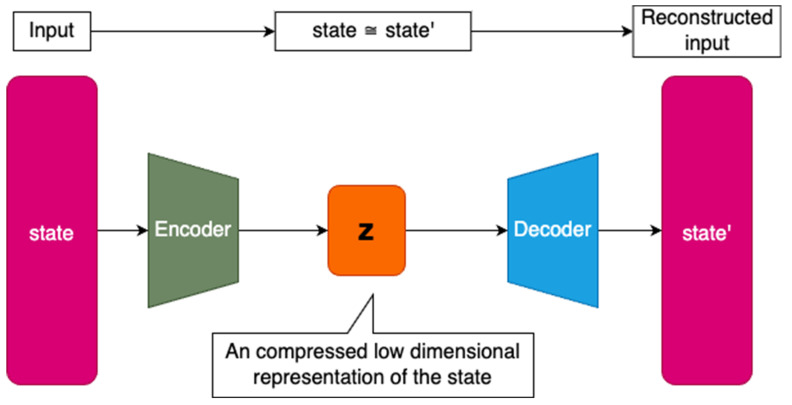
AutoEncoder model with size of the layers indicating the number of neurons in the layers, where the smallest layer **z** in the middle corresponds to low-dimensional latent space representation.

To ensure numerical stability, the average reward is calculated by computing the running mean and standard deviation of the intrinsic reward. This average reward reflects the offset, which is the average error of the AutoEncoder in reconstructing a known signal, and must be subtracted from the intrinsic reward. However, the subtraction can result in negative values, which are not desirable as they may penalize even well-known states. To prevent negative rewards, the Rectified Linear Unit 6 (ReLU-6) function is applied to the normalized intrinsic reward, which ensures that rewards are always positive and prevents the intrinsic reward from becoming too large [34]. The purpose of this reward is to make unvisited states more attractive, rather than to punish frequent visits to a state. For example, in a labyrinth, starting along the same path repeatedly would result in a negative reward, which could lead the agent to consider the path unfavorable. By assigning a reward of 0, the agent considers the path as neutral. The intrinsic reward scale is governed by a standard deviation of 1. The AutoEncoder architecture for anomaly detection is depicted in Figure 1, which takes the state from the environment as input and predicts its reconstruction at the output.

The encoder block of the model is composed of a sequence of fully connected layers, wherein the number of neurons in each layer gradually decreases towards the latent space. The smallest layer in the entire model corresponds to the latent space representation of the encoded state **z**. The decoder block works in the opposite manner, increasing the number of neurons in each fully connected layer towards the output layer. The output layer contains as many neurons as there are features in the agent’s state space. The nonlinearity of the Exponential linear unit (ELU) activation function is used in the hidden layers to facilitate fast and accurate deep neural network learning [35]. Compared to the typically used ReLU activation function, the ELU has an advantage in addressing the dying neuron problem [36]. The model weights are initialized using an Orthogonal initializer with gain set as the square root of 2 [37]. The output layer is activated using a linear function, allowing for an unbounded range of output values and enabling the application of AutoEncoder to different sensor types within a single state space. Similarly, the activation function of the latent space layer **z** is also linear to preserve the unbounded interval property even after state space compression, since this layer is intended to reduce the number of dimensions rather than to purposely limit the interval of compressed values (see Supplementary Materials for source code).

### 2.3. Application of Intrinsic Reward by the AutoEncoder Architecture

Guiding a robot towards signal anomalies can also be used to search a maze efficiently, minimizing the robot’s visits to already-explored areas and guiding it towards unexplored parts. This principle can be compared to a player using a yarn to mark his/her path through the maze. In this case, the memorizing AutoEncoder model represents the yarn, with low reconstruction error indicating that the yarn has been unrolled through here and high error indicating that it hasn’t. This principle can also be applied to more complex problems, as described in Section 2.1, such as landing a lunar lander, balancing an inverted pendulum, swinging a simple 1-joint arm, or driving a car up a hill using acceleration going down the opposite hill.

When combining intrinsic and extrinsic rewards obtained after performing an action, the logic in Table 1 assumes that both signals fall within the range of [0, 1].

There are two possible approaches for merging both reward signals. The first and easier approach is to add up the intrinsic reward and extrinsic reward, where you can regulate the weight of intrinsic reward by modifying the scaling parameter *β*, where *β* > 0 [24].

(4) $$r_{t}=r_{t}^{extrinsic}+\beta \;r_{t}^{intrinsic},$$

A more advanced method involves merging rewards based on their respective *Q* values. This entails generating distinct *Q* values for intrinsic and extrinsic rewards, allowing for the use of specific discount factors (γ) for each predictor [24]. The approach proved beneficial in the Random Network Distillation study, which exhibited the advantage of applying a higher γ for extrinsic *Q* values than for intrinsic *Q* values.

(5) $$Q\left(s,a\right)=Q\left(s,a,\theta^{extrinsic}\right)+\beta \;Q(s,a,\theta^{intrinsic}),$$

The parameters *θ^extrinsic^* and *θ^intrinsic^* represent the *Q* parameters of the extrinsic reward learning model and the *Q* parameters of the intrinsic reward learning model, respectively. Again, the degree of influence of intrinsic reward can be controlled using the *β* parameter.

However, the combination of intrinsic and extrinsic rewards, as shown in Equations (4) and (5), has not been employed in subsequent computations. This is because the purpose was to demonstrate that intrinsic reward alone can guide the agent’s learning process to accomplish the task, even in the absence of extrinsic reward.

This new method of intrinsic rewards does not replace traditional exploration strategies, such as Epsilon-greedy [38] or Boltzmann exploration [39]. Instead, it serves as a complement to these methods, which primarily focus on exploring the agent’s action space. Their role is to strike a balance between purely random action selection and action selection based on predictions made by the neural network from the current state. On the other hand, the intrinsic reward method guides the agent in exploring the state space of the environment, with a focus on the frequency of visiting individual states. By combining both approaches in the future, the agent is expected to gain complete control over the search of the environment.

Like other methods, such as Directed Outreach Reinforcement Action-Selection or Random Network Distillation, AutoEncoder does not reset its knowledge about state visitation between episodes, making it a non-episodic [40] intrinsic reward method. The method memorizes the frequency of state visitation across multiple episodes.

## 3. Results

The agent used in this study was represented by the Dueling Deep Q Network (DQN) algorithm [41], which was chosen due to its suitability for discrete action spaces, encompassing all the test environments used in this paper. DQN has demonstrated success in Atari games [41] and therefore is expected to be capable of solving robot control tasks. The agent employs Boltzmann exploration to search the action space (contrary to the greedy policy), with the temperature parameter linearly decreasing over time using the same decay value until it reaches a preset minimum temperature value. The experiments revealed that extensive searching is advantageous compared to the greedy policy based on learned Q values, as it enables intrinsic reward to attain high values and rewards states that have not been previously observed. Then, a standardized state space [42] can facilitate neural network convergence quickly and accurately. The intrinsic reward can be read from RB or computed directly during DQN update and substituted into Bellman’s equation [43].

Table 2 provides a comprehensive list of the hyperparameters employed in the Acrobot-v1, CartPole-v1, LunarLander-v2, and MountainCar-v0 environments. These hyperparameters were fine-tuned using the W&B Sweeps tool [44], where random search was conducted on 45 combinations of values around the optimal values. The optimal values were identified as the ones that enabled successful completion of the tasks in the aforementioned environments. In Table 2, the abbreviation LS indicates the number of neurons allocated for the latent space, which was determined experimentally to be half the number of features in the state space. In the future, the training process is expected to be accelerated through distributed parallelization [45,46,47].

The cumulative intrinsic and extrinsic rewards obtained by an agent throughout a single episode are represented by the intrinsic and extrinsic scores shown in the following figures. An episode can be defined as a sequence of time steps after which an agent either accomplishes a task or, for example, destroys a robot. If the maximum number of steps for completing a specific task is exceeded, the episode will still come to an external end, but the Markov decision process [48] chain will continue, thanks to the differentiation between the “signal terminated” and “signal truncated” conditions. The “signal terminated” condition refers to the episode ending after reaching a terminal state, as defined by the environment, whereas the “signal truncated” condition refers to the episode ending after an externally set time-limit for solving the task [49].

In cases where the latent space **z** consisted of more than two features, a t-SNE reduction technique [50] was employed to visualize the data in a 2D space. However, when there was only one feature in the latent space, all points were placed on the y-axis with the same value of 1. In instances where there were only two features, the latent space was directly displayed on the graph. In the resulting latent space plots, the points were categorized based on whether the compressed states led directly to a win, making them part of a winning episode, or if they were necessary for exploring the environment, but did not result in a swift win. Two potential scenarios can be inferred from the findings presented below. The first scenario involves the emergence of two distinct clusters when the agent wins even in unseen states. The second scenario occurs when a specific combination of visited states leads to the agent’s victory, and both winning and losing states are concentrated in a single cluster. When a high intrinsic reward is offered, the agent explores entirely new states, whereas even a low intrinsic reward induces an exploration of the action space, ultimately leading to task completion induced by the organization of previously visited states. Consequently, a combination of state-level and action-space searches is crucial.

The novelty intrinsic reward method enabled learning of all tested tasks without the need for external rewards from the environment. The extrinsic scores presented in following figures were solely utilized for evaluating task performance and had no influence on the agent’s learning process (see Supplementary Materials for interactive charts).

**Figure 2:**
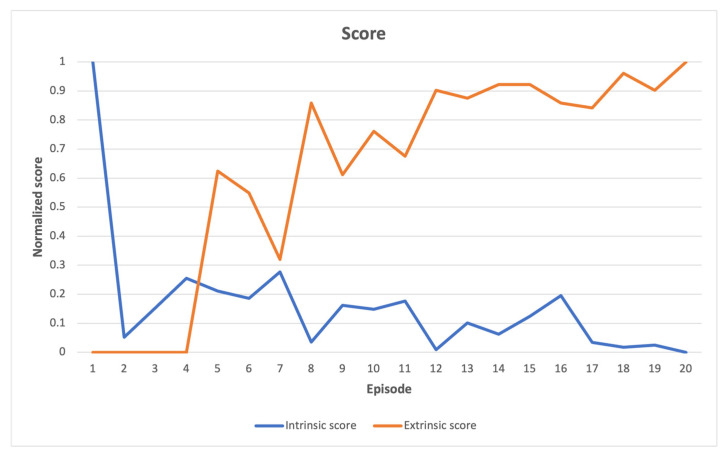
Acrobot-v1 learning scores indicate a relatively steady enhancement in the extrinsic score as the task is completed, which is accompanied by a reduction in novelty exploration. This implies a gradual fine-tuning of parameters.

In Figure 2, a comparison is presented between the normalized intrinsic and extrinsic scores in the Acrobot-v1 environment. This comparison reveals that as the agent explores the environment during learning, the intrinsic scores gradually decrease over time, while the extrinsic score increases. This increase in extrinsic score is due to the agent’s exploration of the environment until it reaches the score threshold required to complete the task, which involves reaching a certain altitude target with its arm. The agent scans multiple altitude levels until it finally reaches the target. The relationship between extrinsic and intrinsic scores demonstrates the interplay between search dynamics and environmental complexity. A high extrinsic score suggests that the task is close to being finished, while a low intrinsic score indicates that only minor adjustments are needed to complete the task, and the learning environment does not present any sudden or unexpected challenges.

**Figure 3:**
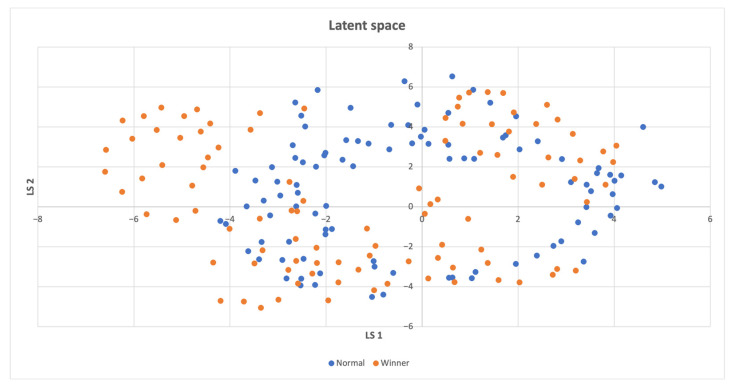
Latent space of the Acrobot-v1 environment, where a distinct cluster consisting of some of the winning states is located on the left.

In Figure 3, the distribution of win points in the latent space is analyzed. The analysis reveals that most of the win points are uniformly distributed over both the latent space and the non-win points, which are frequently recurring states that do not instantly lead to a win. However, there is one small cluster of win points that is distinct from the non-win points.

**Figure 4:**
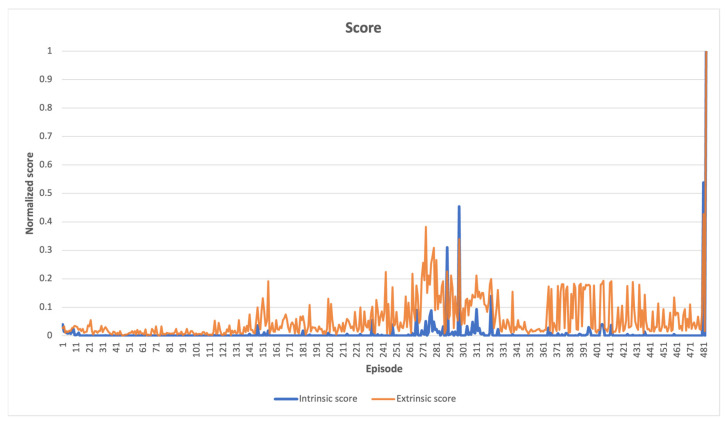
CartPole-v1 learning scores, where both intrinsic and extrinsic fluctuate greatly, indicating the complexity of the search space.

In CartPole-v1 environment, Figure 4 presents a comparison between the normalized intrinsic and extrinsic scores. The comparison reveals that initially, the agent explored the nearby vicinity, which minimized the intrinsic score. As the agent progressed, it encountered novel states leading to an increase in the intrinsic score. Eventually, the agent discovered states that resulted in a win, where both the extrinsic and intrinsic scores were high. The absence of a distinct pattern in the scores indicates that the environment is highly intricate, and making incremental changes is not effective. Instead, progress towards (almost) complete solutions is achieved by exploring previously unexplored areas of the state space.

**Figure 5:**
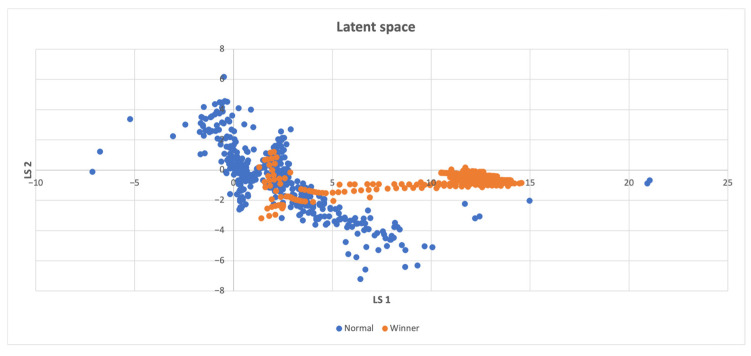
Latent space of the CartPole-v1 environment, the winning states usually differ from the normal ones, implying that discovering novel states is more likely to result in successful completion of the task.

Figure 5 demonstrates that winning points are densely distributed also outside the non-winning points, which implies that these points represent unexplored states.

**Figure 6:**
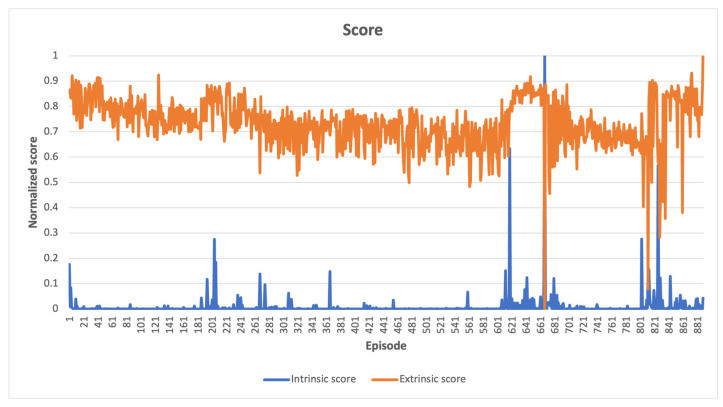
LunarLander-v2 learning scores, high intrinsic values coupled with low extrinsic values around episodes 661 and 800 indicate control failure during exploration of unknown states.

In LunarLander-v2 environment, Figure 6 illustrates a comparison between the intrinsic and extrinsic scores, which have been normalized. The intrinsic score decreases over time as the agent explores its immediate surroundings at the start of learning, while the extrinsic score remains unchanged, indicating no advantage from scanned states. As the agent explores more states, there is a slight increase in the extrinsic score, but the high intrinsic scores are also awarded for states leading to a clear loss, which is not desirable. However, since these states were infrequently visited, it was still possible. At the end of the learning process, the agent solved the task by exceeding the score threshold, but did not achieve a high intrinsic score, which indicates that frequently recurring states leading to a win were observed. The comparison between extrinsic and intrinsic rewards indicates that while the agent may have experienced some significant control failures while exploring uncharted territories, it was able to achieve satisfactory results when the intrinsic reward was high, and the extrinsic reward was low.

**Figure 7:**
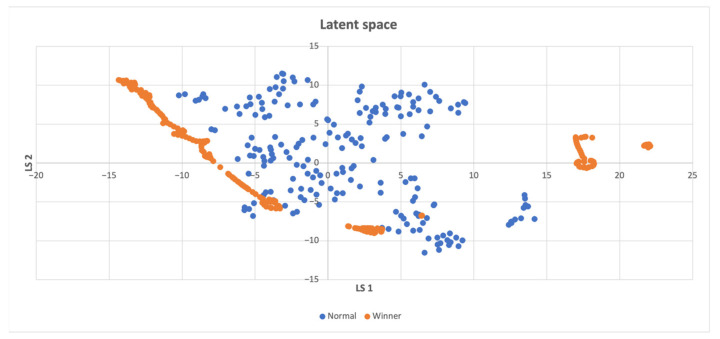
Latent space of the LunarLander-v2 environment, winning states and normal states are seldom close, which implies that unexplored states are crucial for accomplishing the task.

In Figure 7, the win points are clustered separately around the non-win points. The win points mostly consist of previously unvisited states, which suggests that the agent’s exploration was effective in discovering new states.

**Figure 8:**
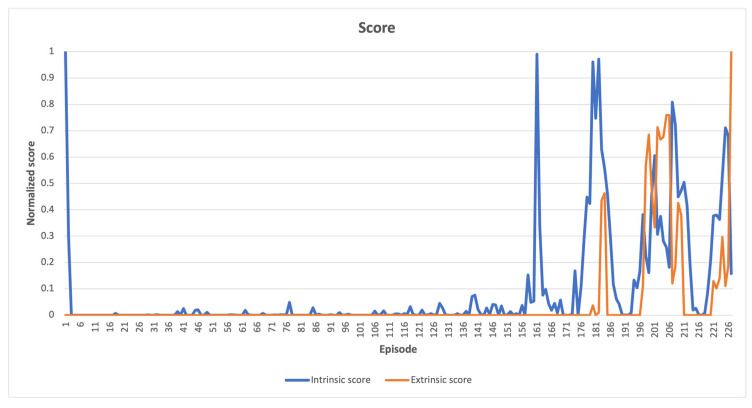
MountainCar-v0 learning scores, as the learning process progresses, newly explored states near the completion of the task yield high scores for both types of measurements.

In MountainCar-v0 environment, Figure 8 presents a comparison between normalized intrinsic and extrinsic scores. The agent apparently scanned its immediate surroundings from the beginning of the learning process. As time progressed, the agent began to uncover novel states, resulting in a high intrinsic score, and the extrinsic score increased gradually. This behavior demonstrates how the car gradually approached the goal state on top of the right hill.

**Figure 9:**
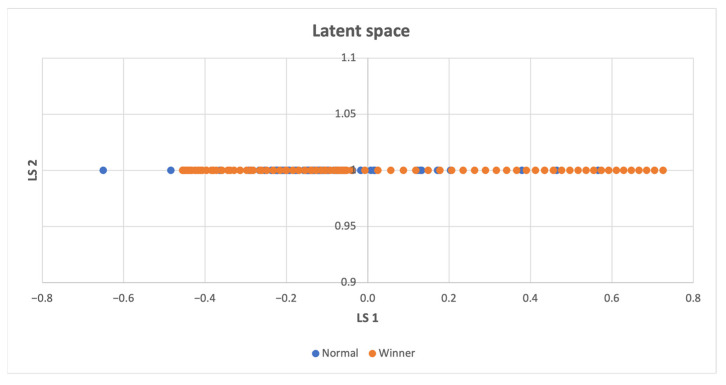
Latent space of the MountainCar-v0 environment, the winning and normal states have a significant overlap, indicating that the completion of the task is unlikely to require abrupt changes in parameters.

Figure 9 illustrates that both win and non-win points are uniformly distributed across the latent space. The frequently repeated states lead to a win.

Table 3 presents a comparison of scores between the approach used in this paper, which solely focused on intrinsic reward, and the conventional DQN results obtained from the CleanRL environment [51] and the Stable-Baselines3 environment [52]. The results in this study were obtained from the average of 100 independent consecutive experiments, and only successful runs that led to solving the given task were included for statistical analysis. The experiment always ended when the agent surpassed a predetermined score threshold [53], indicated in the third column of Table 3. If the agent would continue to search and learn beyond this point, the results would degrade as the winning states would be marked as frequently visited and the agent would explore other inferior directions. The outcomes demonstrate that intrinsic reward alone can be utilized as a search method; however, the agent may not fully understand its true objective in the environment.

One disadvantage of relying solely on intrinsic reward was that, in some instances, the agent got stuck if it exhausted all possibilities in its immediate surroundings, resulting in an average intrinsic reward of 0, which then became a sparse reward.

Despite these potential limitations, the results in Table 3 indicate that, in three out of four environments, using intrinsic reward alone achieved better outcomes than relying solely on extrinsic reward, as shown by the bold scores in the table. In the fourth environment, where intrinsic reward results were slightly inferior, the problem was still solved successfully.

The agents using solely intrinsic reward in all the environments completed the tasks. If the parameters from the previous winning episode were utilized for the agent in a new instance, it would be able to complete the task successfully, as if it had learned from an external reward. However, the caveat is that without the user setting a score threshold or some other indicator, relying solely on intrinsic reward would not provide any indication of task completion, and agent would continue searching.

### When to Compute the Intrinsic Reward

There are two possible approaches to calculating intrinsic rewards. One approach involves expressing the intrinsic reward after the agent has obtained the state from the environment and storing it in the experience replay buffer (RB). In this case, the AutoEncoder is trained on the observed state_t_ from the RB, while the intrinsic reward prediction is made based on the current state_t_ used by the agent in the environment to predict the action_t_. However, this can result in off-policyness, where intrinsic rewards sampled from the past may no longer correspond to the current visitation frequency of a given state and inaccurately affect the agent’s policy during DQN model updates. The approach in “Never Give Up” [55] also stores the intrinsic reward in RB.

Alternatively, intrinsic rewards can be computed during the update of the DQN model without immediately imposing the reward. Since states are usually part of the stored experiences, intrinsic rewards can be computed based on the current state visitations in the agent’s state space. In this case, the AutoEncoder is taught on the state_t_ from RB, and the intrinsic reward prediction is done from state_t+1_, which is also part of RB. To compare both options, statistics were generated in different environments with 100 runs in each case.

According to Table 4, the optimal timing for calculating the intrinsic reward is dependent on the type of environment and the specific task being performed. For the selected environments, MountainCar-v0 and LunarLander-v2 exhibited more consistent goal convergence when the intrinsic reward was computed during DQN model updates, using the stored states in RB. On the other hand, for the CartPole-v1 environment, it was more advantageous to store intrinsic rewards in RB and then sample them during DQN model updates. Despite the difficulty in learning due to sparse extrinsic rewards, MountainCar-v0 achieved a high task success rate of up to 98 percent when intrinsic rewards were utilized.

## 4. Discussion

In the present study, extrinsic reward was not employed. Instead, the paper demonstrates the effectiveness of novelty-seeking intrinsic rewards for reinforcement learning, rendering extrinsic rewards unnecessary. Despite the absence of extrinsic reward, the task were successfully completed, most of them even better than when extrinsic rewards were tested in other studies [51,54]. Nonetheless, it is recommended that future robot learning should incorporate a fusion of both intrinsic and extrinsic rewards for optimal results. The blending of intrinsic reward-based robot control with existing control techniques described earlier in Equations (4) and (5) combines two different approaches to controlling robots in a way that leverages the strengths of each approach. In this way, the robot is able to take advantage of the stability and reliability of existing control techniques, while also benefiting from the flexibility and adaptability of intrinsic reward-based control. In future, more advanced methods of blending various types of rewards can be explored.

The method proposed here allows a robot to explore its immediate surroundings during an autonomous search by detecting anomalies that guide it towards uncharted terrain. For instance, a Mars rover that lacks real-time communication with Earth might use the proposed method instead of the currently used grid-based planner approach. The presently employed planner generates a terrain map using stereo cameras and creates a path on the grid using the Field D* planning algorithm [56]. This algorithm assigns a cost to each grid cell and generates a path that minimizes this cost. However, this paper proposes a different approach that utilizes a neural network to generate paths based on previously unseen states. The goal is to choose a path that provides the newest information while exploring the Martian surface, rather than selecting the most optimal path.

Another example where the robot’s success depends on discovering unexplored terrain is archaeological exploration of the seabed or land at greater depths. Typically, search techniques such as the random tree algorithm (RRT) or RRT* are used to navigate empty areas quickly and locate the next traversable route in complex environments [57]. In contrast, this paper focuses on using a pure neural network as a memory instead of tree generation. This approach can also be useful in natural disaster response by automating search in complex environments and guiding the robot to a target or exit. In each of these scenarios, the intrinsic reward needs to be supplemented with information about what the robot should search for.

Intrinsic reward can help to guide the robot’s behavior also in other ways. For example, the robot could be rewarded for achieving a certain level of accuracy in its movements or to encourage the robot to perform the task more quickly or using less energy. The introduction stated that such intrinsic reward applications have been previously employed successfully in other contexts without the use of an autoencoder, which is utilized in the present study. It is suggested that using an autoencoder for intrinsic reward may be more suitable for more intricate tasks such as novelty search.

## 5. Conclusions

The results successfully demonstrated how intrinsic rewards using autoencoder for multiple signal input processing can aid in navigating an agent through the state search space representing robot’s motion within its environment. Such a capability could prove useful in scenarios where a robot has limited means of communication with its human operator. This should be beneficial when a robot needs to operate in environments that are hazardous or difficult for humans to access. In subsequent research, an operational robot will be deployed to evaluate the effectiveness of intrinsic rewards in resolving the same tasks performed by the simulated robots. It should provide insights into the real-world application of intrinsic rewards in robotics and could lead to the development of more advanced and efficient autonomous systems.

## Figures and Tables

**Table 1:** The logic of combining intrinsic with extrinsic reward elucidated through examples of extreme value combinations and the novelty of the current state in relation to task completion, as depicted in further figures.

Intrinsic Reward	Extrinsic Reward	Result
0	0	A frequently recurring state leading to a loss
1	0	New unseen state leading to a loss
0	1	Frequently recurring state leading to a win
1	1	New unseen state leading to a win

**Table 2:** Optimized hyperparameters for Dueling DQN agent.

Name	Description	Value
Epochs	Number of training episodes	2000
Buffer size	The capacity of experience replay buffer	1,000,000
Min. temperature	The minimal value of temperature for Boltzmann exploration	0.01
Init. temperature	The maximal value of temperature for Boltzmann exploration	1.0
Decay temperature	The value of temperature reduction	1 × 10^−5^
Batch size	Number of samples applied during training at once	256
Learning rate	The learning rate for training process	3 × 10^−4^
Global clipnorm	Clipping applied globally on gradients	1.0
τ	Soft target update value for coping original to target DQN	0.01
γ	Discount factor for Bellman equation	0.99
No. neurons in DQN	Number of neurons for each hidden layer	512, 256
No. neurons in AutoEncoder	Number of neurons for each hidden layer	128, 64, 32, 16, LS, 16, 32, 64, 128

**Table 3:** The comparison of DQN’s performance using solely extrinsic reward and intrinsic reward. The results are evaluated based on the score, where a more positive score signifies better performance. The task’s completion requires surpassing the score threshold in the last column, and both approaches attained it for all environments. The superior outcomes are highlighted in bold.

Environment	Score (w/Extrinsic Reward Only)	Score (This Paper)	Score Threshold
MountainCar-v0	−194.95 ± 8.48 [51]	**−96.684 ± 7.028**	−110
Acrobot-v1	−91.54 ± 7.20 [51]	**−86.12 ± 4.604**	−100
CartPole-v1	488.69 ± 16.11 [51]	**499.483 ± 3.05**	475
LunarLander-v2	**280.22 ± 13.03** [54]	234.881 ± 33.86	200

**Table 4:** Problem-solving success in all tested environments for different methods of updating the intrinsic score. No single method can be considered universally better, as the effectiveness of each method depends on the specific environment. Best results are highlighted in bold.

Environment	Method	Percentage Success Rate
Acrobot-v1	Stored in RB	100
	Calculated during DQN model update	100
CartPole-v1	Saved in RB	**75**
	Calculated during DQN model update	60
LunarLander-v2	Saved in RB	11
	Calculated during DQN model update	**14**
MountainCar-v0	Saved in RB	61
	Calculated during DQN model update	**98**

## Data Availability

Not applicable.

## Supplementary Materials

Interactive charts: https://wandb.ai/markub/Dueling-DQN-with-AutoEncoder (accessed on 2 April 2023); Source codes: https://github.com/markub3327/Dueling-DQN-with-AutoEncoder (accessed on 2 April 2023).

## References

[1] Pathak D., Agrawal P., Efros A.A., Darrell T. Curiosity-Driven Exploration by Self-Supervised Prediction. Proceedings of the International Conference on Machine Learning, PMLR.

[2] Burda Y., Edwards H., Storkey A., Klimov O. (2018). Exploration by Random Network Distillation. https://arxiv.org/abs/1810.12894.

[3] Bellemare M., Srinivasan S., Ostrovski G., Schaul T., Saxton D., Munos R. (2016). Unifying Count-Based Exploration and Intrinsic Motivation. Adv. Neural Inf. Process. Syst..

[4] Tang H., Houthooft R., Foote D., Stooke A., Xi Chen O., Duan Y., Schulman J., DeTurck F., Abbeel P. (2017). # Exploration: A Study of Count-Based Exploration for Deep Reinforcement Learning. Adv. Neural Inf. Process. Syst..

[5] Oudeyer P.Y., Kaplan F., Hafner V.V. (2007). Intrinsic motivation systems for autonomous mental development. IEEE Trans. Evol. Comput..

[6] Houthooft R., Chen X., Duan Y., Schulman J., De Turck F., Abbeel P. (2016). Vime: Variational Information Maximizing Exploration. Adv. Neural Inf. Process. Syst..

[7] Choshen L., Fox L., Loewenstein Y. (2018). Dora the Explorer: Directed Outreaching Reinforcement Action-Selection. https://arxiv.org/pdf/1804.04012.pdf.

[8] Kamar D., Üre N.K., Ünal G. (2022). GAN-based Intrinsic Exploration for Sample Efficient Reinforcement Learning. https://arxiv.org/pdf/2206.14256.pdf.

[9] Kamalova A., Lee S.G., Kwon S.H. (2022). Occupancy Reward-Driven Exploration with Deep Reinforcement Learning for Mobile Robot System. Appl. Sci..

[10] Liu Q., Liu Z., Xiong B., Xu W., Liu Y. (2021). Deep reinforcement learning-based safe interaction for industrial human-robot collaboration using intrinsic reward function. Adv. Eng. Inform..

[11] Chen Z., Subagdja B., Tan A.H. End-to-End Deep Reinforcement Learning for Multi-Agent Collaborative Exploration. Proceedings of the 2019 IEEE International Conference on Agents (ICA).

[12] Shi H., Shi L., Xu M., Hwang K.S. (2019). End-to-end navigation strategy with deep reinforcement learning for mobile robots. IEEE Trans. Ind. Inform..

[13] Nguyen T., Luu T.M., Vu T., Yoo C.D. Sample-Efficient Reinforcement Learning Representation Learning with Curiosity Contrastive forward Dynamics Model. Proceedings of the 2021 IEEE/RSJ International Conference on Intelligent Robots and Systems (IROS).

[14] Zhang C., Ma L., Schmitz A. (2020). A sample efficient model-based deep reinforcement learning algorithm with experience replay for robot manipulation. Int. J. Intell. Robot. Appl..

[15] Burgueño-Romero A.M., Ruiz-Sarmiento J.R., Gonzalez-Jimenez J. (2021). Autonomous Docking of Mobile Robots by Reinforcement Learning Tackling the Sparse Reward Problem. Advances in Computational Intelligence: 16th International Work-Conference on Artificial Neural Networks, IWANN 2021, Virtual Event. Proceedings, Part II.

[16] Huang S.H., Zambelli M., Kay J., Martins M.F., Tassa Y., Pilarski P.M., Hadsell R. (2019). Learning gentle object manipulation with curiosity-driven deep reinforcement learning. arXiv.

[17] Szajna A., Kostrzewski M., Ciebiera K., Stryjski R., Woźniak W. (2021). Application of the Deep CNN-Based Method in Industrial System for Wire Marking Identification. Energies.

[18] Hessel M., Modayil J., Van Hasselt H., Schaul T., Ostrovski G., Dabney W., Horgan D., Piot B., Azar M., Silver D. (2018). Rainbow: Combining Improvements in Deep Reinforcement Learning. Proc. AAAI Conf. Artif. Intell..

[19] Pang G., van den Hengel A., Shen C., Cao L. Toward Deep Supervised Anomaly Detection: Reinforcement Learning from Partially Labeled Anomaly Data. Proceedings of the 27th ACM SIGKDD Conference on Knowledge Discovery & Data Mining.

[20] Michalski P. (2021). Anomaly Detection in the Context of Reinforcement Learning. https://www.researchgate.net/profile/Patrik-Michalski/publication/354694975_Anomaly_detection_in_the_context_of_Reinforcement_Learning/links/6148336fa595d06017db791d/Anomaly-detection-in-the-context-of-Reinforcement-Learning.pdf.

[21] Wang Y., Xiong L., Zhang M., Xue H., Chen Q., Yang Y., Tong Y., Huang C., Xu B. Heat-RL: Online Model Selection for Streaming Time-Series Anomaly Detection. Proceedings of the Conference on Lifelong Learning Agents.

[22] Ma X., Shi W. (2020). Aesmote: Adversarial Reinforcement Learning with Smote for Anomaly Detection. IEEE Trans. Netw. Sci. Eng..

[23] Rafati J., Noelle D.C. (2019). Learning Representations in Model-Free Hierarchical Reinforcement Learning. Proc. AAAI Conf. Artif. Intell..

[24] Badia A.P., Piot B., Kapturowski S., Sprechmann P., Vitvitskyi A., Guo Z.D., Blundell C. Agent57: Outperforming the Atari Human Benchmark. Proceedings of the 37th International Conference on Machine Learning, ICML 2020, PMLR.

[25] Lindegaard M., Vinje H.J., Severinsen O.A. (2023). Intrinsic Rewards from Self-Organizing Feature Maps for Exploration in Reinforcement Learning. arXiv.

[26] Brockman G., Cheung V., Pettersson L., Schneider J., Schulman J., Tang J., Zaremba W. (2016). Openai Gym. arXiv.

[27] Barto A.G., Sutton R.S., Anderson C.W. (1983). Neuronlike aDaptive Elements That Can Solve Difficult Learning Control Problems. IEEE Trans. Syst. Man Cybern..

[28] Sutton R.S. (1995). Generalization in Reinforcement Learning: Successful Examples Using Sparse Coarse Coding. Adv. Neural Inf. Process. Syst..

[29] Sutton R.S., Barto A.G. (2018). Reinforcement Learning: An Introduction.

[30] Moore A.W. (1990). Efficient Memory-Based Learning for Robot Control.

[31] Jakovlev S., Voznak M. (2022). Auto-Encoder-Enabled Anomaly Detection in Acceleration Data: Use Case Study in Container Handling Operations. Machines.

[32] Fedus W., Ramachandran P., Agarwal R., Bengio Y., Larochelle H., Rowland M., Dabney W. Revisiting Fundamentals of Experience Replay. Proceedings of the 37th International Conference on Machine Learning, ICML 2020, PMLR.

[33] Feeney P., Hughes M.C. (2021). Evaluating the Use of Reconstruction Error for Novelty Localization. https://arxiv.org/pdf/2107.13379.pdf.

[34] Krizhevsky A. (2010). Convolutional Deep Belief Networks on Cifar-10. http://www.cs.utoronto.ca/%7Ekriz/conv-cifar10-aug2010.pdf.

[35] Clevert D.A., Unterthiner T., Hochreiter S. (2015). Fast and Accurate Deep Network Learning by Exponential Linear Units (Elus). https://arxiv.org/pdf/1511.07289v5.pdf.

[36] Lu L., Shin Y., Su Y., Karniadakis G.E. (2019). Dying Relu and Initialization: Theory and Numerical Examples. https://arxiv.org/pdf/1903.06733.pdf.

[37] Saxe A.M., McClelland J.L., Ganguli S. (2013). Exact Solutions to the Nonlinear Dynamics of Learning in Deep Linear Neural Networks. https://arxiv.org/pdf/1312.6120.pdf.

[38] Mnih V., Kavukcuoglu K., Silver D., Graves A., Antonoglou I., Wierstra D., Riedmiller M. (2013). Playing Atari with Deep Reinforcement Learning. https://arxiv.org/pdf/1312.5602v1.pdf.

[39] Usama M., Chang D.E. Learning-Driven Exploration for Reinforcement Learning. Proceedings of the 2021 21st International Conference on Control, Automation and Systems (ICCAS).

[40] Steinparz C.A. (2021). Reinforcement Learning in Non-Stationary Infinite Horizon Environments/submitted by Christian Alexander Steinparz. BSc. Master’s Thesis.

[41] Wang Z., Schaul T., Hessel M., Hasselt H., Lanctot M., Freitas N. Dueling Network Architectures for Deep Reinforcement Learning. Proceedings of the International Conference on Machine Learning.

[42] LeCun Y., Bottou L., Bengio Y., Haffner P. (1998). Gradient-based learning applied to document recognition. Proc. IEEE.

[43] Jang B., Kim M., Harerimana G., Kim J.W. (2019). Q-learning algorithms: A comprehensive classification and applications. IEEE Access.

[44] Weights & Biases: Tune Hyperparameters. https://docs.wandb.ai/guides/sweeps.

[45] Zhao X., An A., Liu J., Chen B.X. Dynamic Stale Synchronous Parallel Distributed Training for Deep Learning. Proceedings of the 2019 IEEE 39th International Conference on Distributed Computing Systems (ICDCS).

[46] Šimon M., Huraj L., Siládi V. (2013). Analysis of performance bottleneck of P2P grid applications. J. Appl. Math. Stat. Inform..

[47] Skrinarova J., Dudáš A. (2022). Optimization of the Functional Decomposition of Parallel and Distributed Computations in Graph Coloring With the Use of High-Performance Computing. IEEE Access.

[48] Van Otterlo M., Wiering M. (2012). Reinforcement Learning and Markov Decision Processes. Reinforcement Learning: State-of-the-Art.

[49] Pardo F., Tavakoli A., Levdik V., Kormushev P. Time Limits in Reinforcement Learning. Proceedings of the International Conference on Machine Learning.

[50] Van der Maaten L., Hinton G. (2008). Visualizing Data Using t-SNE. J. Mach. Learn. Res..

[51] Huang S., Dossa R.F.J., Ye C., Braga J., Chakraborty D., Mehta K., Araújo J.G. (2021). CleanRL: High-quality Single-File Implementations of Deep Reinforcement Learning Algorithms. https://www.jmlr.org/papers/volume23/21-1342/21-1342.pdf.

[52] Raffin A., Hill A., Gleave A., Kanervisto A., Ernestus M., Dormann N. (2021). Stable-baselines3: Reliable reinforcement learning implementations. J. Mach. Learn. Res..

[53] Balis J. Gymnasium. https://github.com/Farama-Foundation/Gymnasium/blob/main/gymnasium/envs/__init__.py.

[54] Raffin A. DQN Agent Playing LunarLander-v2. https://huggingface.co/araffin/dqn-LunarLander-v2.

[55] Badia A.P., Sprechmann P., Vitvitskyi A., Guo D., Piot B., Kapturowski S., Tieleman O., Arjovsky M., Pritzel A., Bolt A. (2020). Never Give Up: Learning Directed Exploration Strategies. https://arxiv.org/abs/2002.06038.

[56] Carsten J., Rankin A., Ferguson D., Stentz A. Global Path Planning on Board the Mars Exploration Rovers. Proceedings of the 2007 IEEE Aerospace Conference.

[57] Liu J. (2022). Research on the Development and Path Exploration of Autonomous Underwater Robots. ITM Web Conf..

